# Relations Between Bilingualism and Autistic-Like Traits in a General Population Sample of Primary School Children

**DOI:** 10.1007/s10803-019-03994-2

**Published:** 2019-04-11

**Authors:** Draško Kašćelan, Napoleon Katsos, Jenny L. Gibson

**Affiliations:** 1 Faculty of Modern and Medieval Languages, University of Cambridge, Raised Faculty Building, Sidgwick Avenue, Cambridge, CB3 9DA UK; 2 Faculty of Education, University of Cambridge, 184 Hills Road, Cambridge, CB2 8PQ UK

**Keywords:** Autistic-like traits, Autism, Bilingualism, Structural language, Child language

## Abstract

Some evidence suggests that bilingualism improves communication and cognitive skills which are often impaired in autism. However, diagnosing autism in bilinguals may suffer a cultural bias, which can affect the investigation of bilingualism and autism. Therefore, the current study investigates relations between autistic-like traits (ALTs) and bilingualism in a general population sample of 394 children (M age = 8;3). Within the high-scoring group on the ALT measure, monolinguals had significantly higher ALT scores than bilinguals. There were no differences between monolinguals and bilinguals in the low-scoring group. Across the whole sample, age and structural language skills accounted for 35% variance in ALTs, while bilingualism had no effect on ALTs. Furthermore, structural language skills explained more variance in ALTs among bilinguals than among monolinguals.

**Electronic supplementary material:**

The online version of this article (10.1007/s10803-019-03994-2) contains supplementary material, which is available to authorized users.

## Introduction

Research into autism spectrum disorders (ASD) has identified some areas of development which present challenges for many individuals with this condition, although their presence is not a requirement for meeting diagnostic criteria. These include deficits in specific pragmatic skills (Lam and Yeung 2012), executive control (Schuh and Eigisti 2012) and Theory of Mind (Baron-Cohen et al. 2000). Interestingly, many of these same areas have been identified as strengths in certain bilingual contexts (e.g., Siegal et al. 2010; Carlson and Meltzoff 2008; Kovács 2009). This has prompted a number of studies investigating bilingualism in populations with ASD (see Uljarević et al. 2016). However, the heterogeneity of both bilingualism and ASD brings several methodological challenges. In this paper, we point out that ASD diagnosis may be subject to cultural bias, especially in bilinguals. Rather than relying on a distinction between clinical and neurotypically developing groups, we investigate the interaction between autistic-like traits (ALTs) and bilingualism in a general population sample. Apart from considering participants’ language status (monolingual or bilingual) in relation to ALTs, among the bilingual subsample we aim to investigate if the nature of their bilingualism (e.g., proficiency and literacy in both languages, length of exposure to both languages, etc.) affects variability in their ALTs.

We first provide a brief rationale for investigating the interaction between bilingualism and ASD, followed by a review of previous studies on the topic. Second, we discuss the difficulties of diagnosing ASD in bilinguals due to cultural and gender bias. Next, we outline the work on autistic traits which has been done in the general population. This will lead to the outline of the present study and research questions to be investigated.

## Bilingualism and ASD

A large body of literature has shown bilingual advantages in specific cognitive and communicative domains, such as in executive functions (Carlson and Meltzoff 2008; Poarch and van Hell 2012; Kroll and Bialystok 2013; Barac et al. 2014), Theory of Mind (Goetz 2003; Kovács 2009; Nguyen and Wilde Astington 2014; Rubio-Fernández and Glucksberg 2012), and pragmatic skills (Siegal et al. 2010; Antoniou and Katsos 2017; Lorge and Katsos 2018). However, some recent studies found no difference between monolinguals and bilinguals in cognitive skills (Antón et al. 2014; Duñabeita et al. 2014; Dahlgren et al. 2017). These discrepancies in findings suggest that due to the heterogeneity of bilingual experiences, bilingual advantages do not emerge in every bilingual context (Bak 2016; Valian 2015a, b). As pointed out by Bialystok and Grundy (2018), positive evidence regarding bilingual advantages should not be categorically dismissed but rather prompt further engagement with the topic.

Meanwhile, these same areas can often be impaired in ASD. In addition to the two core deficits of autism, related to social communication and interaction, and restricted and repetitive patterns of interests or activities (American Psychiatric Association [APA] 2013, p. 53), individuals with ASD may show impairment in executive functions (Schuh and Eigisti 2012; Brady et al. 2013), Theory of Mind (Baron-Cohen 1989, 1991), and some aspects of pragmatics (Lam and Yeung 2012). Therefore, the research above suggests that some areas that are commonly challenging for individuals with ASD can be areas of strength for bilinguals. This raises intriguing questions about the interaction of bilingual and autistic cognition. For example, could bilingualism influence some of the cognitive and communicative difficulties associated with autism? Alternatively, does bilingual development, which can be accompanied by a lag in receptive vocabulary acquisition in the early years of life (Hoff et al. 2012; Bialystok et al. 2010), impose an additional burden on the development of individuals with ASD?

Several studies have compared early language and cognitive development of bilinguals and monolinguals with ASD. For instance, Petersen et al. (2012) compared 14 Chinese-English bilinguals to 14 English monolinguals on receptive language (Peabody Picture Vocabulary Test-Third Edition [PPVT-III], Dunn and Dunn 1997), comprehension and production skills (The Preschool Language Scale, Zimmerman et al. 1992), nonverbal IQ (The Mullen Scales of Early Learning, Mullen 1995) and parental assessment of their children’s language ability (The Communicative Development Inventories, Fenson et al. 1993). Bilinguals did not lag behind monolinguals. In fact, bilinguals’ conceptual vocabulary (sum of familiar concepts in both languages without translation equivalents) and English vocabulary size were non-significantly larger than in monolinguals, which was driven by bilinguals’ significantly higher non-verbal IQ. Considering communication skills, Reetzke et al. (2015) found no difference between a group of 23 bilinguals and 31 monolinguals on communication scores as measured by Children’s Communication Checklist-2 (CCC-2, Bishop 2003). A review by Uljarević et al. (2016) supported these findings by concluding that bilinguals with ASD do not seem to lag behind autistic monolinguals in language development. Furthermore, bilingual advantage has been found in cognitive skills, as shown by a recent study by Gonzalez-Barrero and Nadig (2017). Specifically, they reported that a small group of bilingual children with ASD (n = 10, age M = 97 months, *SD* = 7.23) outperformed monolinguals with ASD (n = 10, age M = 100 months, *SD* = 11.94) on a set-shifting measure.

While these and other studies have looked at language and some aspects of executive functions, no research to date has investigated comprehensively the potential effect of bilingualism on the range of behaviours that characterize the autistic profile. However, before such an investigation is launched, it is important to bear in mind that studies looking into the recognition of ASD symptoms in different cultures and communities show cultural and gender bias in diagnosis (Matson et al. 2017; Burke et al. 2015; Lai et al. 2015). Such a bias is particularly likely to arise with bilingual populations.

## ASD Symptoms and Bilingualism

Recent studies suggest that culture and gender norms can affect the recognition of ASD symptoms in parental reports or in the specialists’ observations. For instance, in a study comparing parental ratings of autistic children’s behaviour to same-aged peers in a sample of Greek, Italian, Japanese, Polish, and US children, Matson et al. (2017) found that the interpretation of restricted and repetitive behaviour, one of the two core areas of impairment in ASD, seems to be culturally subjective. Bilinguals often grow up to be bicultural through exposure to and use of their two languages, although with some exceptions (Grosjean 2015). Consequently, there is a question whether autistic traits can be recognised in bilinguals in the same way as in monolinguals due to potential cultural biases. Burke et al. (2015) found that school-based professionals in the Netherlands identify autistic behaviour more often in children with Dutch background (72%) than in children with English and French background (48%) or in those with Moroccan and Turkish background (44%). Therefore, by looking only at bilinguals with a clinical diagnosis of ASD, research studies risk excluding a large number of bilingual individuals who have not received a diagnosis due to cultural bias in the recognition of ASD symptoms.

Additionally, Goldman (2013) and Lai et al. (2015) suggest that the perception of autistic symptoms seems to be affected by gender.^1^ That is, in cases of the same social deficits, a female child might be simply considered shy, while boys will be considered unresponsive. Sutherland et al. (2017) found that girls’ specific and detailed interests in reading, arts/crafts or singing/dancing/music follow traditional gender norms, which may leave them unmarked by specialists/clinicians as indicators of ASD. Since gender norms are often driven by cultural standards and expectations, this poses an additional difficulty in acknowledging ASD symptoms in bicultural bilinguals as compared to monolinguals.

As these studies suggest, investigating bilingualism solely in the clinical population with ASD is complicated by the possible diagnostic bias caused by cultural and gender norms. In the next section, we propose a novel approach to address this issue by looking at autistic-like traits and bilingualism in a broader way.

## Autistic-Like Traits and the Current Study

The concept of ‘autistic-like’ traits (ALTs) in the general population represents a part of a broader ASD phenotype (Baron-Cohen et al. 2001). Specifically, ALTs include difficulties in social communication/interaction and restricted interests or repetitive behaviours, which lie on a continuum below the clinical threshold. These traits have been extensively studied in the general population monolingual samples (Constantino and Todd 2003; Ronald et al. 2005; Armstrong et al. 2017). For instance, a study by Constantino and Todd (2003) looked at autistic-like traits in a group of 788 twin pairs (age range 7–15 years). ALTs were measured by using the Social Responsiveness Scale ([SRS] Constantino 2002), a 65-item parent and/or teacher questionnaire examining autistic symptoms quantitatively. The authors found that in the general population, autistic-like traits are: ‘(1) common; (2) continuously distributed; (3) moderately to highly heritable; (4) influenced by the same additive genetic factors in boys and girls; and (5) exhibit no evidence of nonadditive genetic factors’ (pp. 527–528). A similar approach of investigating autistic-like traits was taken in Haraguchi et al. (2018). In this longitudinal study, ALTs were measured in 168 Japanese children (89 males) by using the Japanese version of SRS. The study found that in both boys and girls, ALTs are stable between the ages of 5 and 8. Furthermore, the study offered reliable support to the approaches that investigate autistic-like traits as a continuous variable in general population samples. In addition to SRS, other self-report and parental questionnaires, such as, for instance, the autism-spectrum quotient (AQ), have reliably been used in the general population samples to quantify ALTs (see Baron-Cohen et al. 2001; Armstrong et al. 2017).

Considering this continuous distribution of ALTs in the general population, we are able to study the interplay between bilingualism and ALTs on a much larger scale rather than solely looking into the population diagnosed with ASD. Investigating ALTs in a general population can offer an important complementary insight into the nature of these traits and their prevalence in both monolinguals and bilinguals, without being subject to cultural or other bias (since an autism diagnosis is irrelevant to the study). Therefore, in the current study, we investigate the interaction between bilingualism and autistic-like traits by using a general population approach. Instead of comparing a group with a clinical diagnosis of ASD to a group with neurotypical development, we compare individuals who are high-scorers vs. individuals who are low-scorers on a measure of autistic-like traits. Furthermore, by looking at a large sample of children with various levels of ALTs, we aim to investigate explanatory factors of ALTs among both bilinguals and monolinguals. This approach allows us to overcome biases of differential rates of clinical diagnosis between bilingual and monolingual groups caused by cultural differences or gender stereotypes. To our knowledge, this is the first study to investigate bilingualism and ALTs using this approach.

The groups of interest in this study are monolingual and bilingual primary school children in the United Kingdom (UK). The aim of the study is to answer the following research questions:

1. Is there a difference in the proportion of monolinguals and bilinguals found in groups selected from the extremes (high and low) of the autistic-like traits distribution?
2. Within the high and low-scoring groups identified in (1), do bilinguals and monolinguals differ in average ALT scores?
3. What factors account for observed variance in ALT scores in a general population sample of children?
4. Considering the complexities of bilingual experiences (e.g., proficiency in both languages, use of languages), what factors account for observed variance in ALT scores in the bilingual subsample?

## Methods

### Ethics

Ethical review and permissions were obtained from the Institutional ethics committee. As participants about whom the data was collected were minors, parents/caregivers gave informed written consent on their behalf.

### Sampling and Recruitment

A call for participation was sent to 333 state primary schools in the local area, or within reasonable travelling distance for the researchers gathering data (Cambridgeshire and London). Fourteen schools accepted participation. All caregivers and their children were invited via the schools to take part in the study. All primary school children were eligible for the study (common age range in UK primary schools: 5–12 years). Caregivers were informed that the three schools with the highest response rate relative to the number of students would receive book vouchers for the school library in the value of £150, £100, and £50. Data was collected on 394 children.

### Measures

A questionnaire pack was sent to caregivers comprising a consent form and the following three questionnaires: language use and socioeconomic status (SES) questionnaire, social skills improvement system (SSIS) rating scales (Gresham and Elliott 2008), and Children’s Communication Checklist ([CCC-2], Bishop 2003).

#### Language Use and SES Questionnaire

This questionnaire was formed based on the Alberta language environment questionnaire (Paradis 2011), and the family affluence scale (Currie et al. 2008) as used in Antoniou and Katsos (2017) with some additional modifications. The questionnaire included five sections: (1) demographic information about the child, (2) information about the child’s language abilities and exposure, (3) information about the caregivers’ use of language(s) with the child, (4) information about the family, and (5) information about the child’s and other family members’ difficulties (if any). Child’s language skills (speaking, understanding, writing, and reading) for every language separately were rated on a 5-point Likert scale, 1 being *not competent* and 5 being *very competent*. For language use (range 0–1), values larger than 0.5 indicate more use of English with caregivers, while values smaller than 0.5 indicate more use of other language(s) (see Paradis 2011 for more details on this measure). Caregivers’ educational levels were scored as follows: 1 for primary school, 2 for secondary school or other qualifications, 3 for a bachelor’s degree, 4 for a master’s degree, and 5 for a doctoral degree.

#### Social Skills Improvement System-Rating Scales (SSIS-RS)

SSIS-RS Parent Form is a questionnaire designed for parents to assess children’s social skills and problem behaviours in both typically and atypically developing children. Parents are asked to rate 46 statements about their child’s social skills and 33 statements regarding their child’s problem behaviours on a 4-point scale, indicating how often the child behaves in a certain way (never, seldom, often, or almost always). The questionnaire taps into various social skills (e.g., communication, co-operation, empathy, etc.) and competing problem behaviours (e.g. externalising, bullying, hyperactivity/inattention, autism spectrum, etc.). Of relevance for this study is the subscale providing autism spectrum score, obtained from responses on 15 statements in the questionnaire (8 from the social skills subset, and 7 from the problem behaviours section). The manual offers a classification of autistic traits raw scores into three categories (below average, average, above average) for primary school children, which matches the profile of our participants. The autism spectrum raw score can range from 0 to 45, with higher scores indicating higher autistic traits. For the age group between 5 and 12 years, raw scores from 0 to 2 indicate − 1SD from the population average or more, and form the below average group. Raw scores from 3 to 14 present average population scores, while raw scores above 15 indicate above average levels of autistic traits (i.e., +1SD from the population mean or more). For further details on the standardisation of SSIS-RS see Gresham and Elliott (2008).

#### Children’s Communication Checklist (CCC-2)

This 70-item parental questionnaire quantifies the communication skills of children between the ages of 4 and 16 years. The questionnaire yields the following standard scores for each child in relation to their communication skills: (a) speech, (b) syntax, (c) semantics, (d) coherence, (e) inappropriate initiation, (f) stereotyped language, (g) use of context, (h) nonverbal communication, (i) social relations, and (j) interests. Furthermore, the following scores can be derived: (1) the general communication composite (GCC, sum of a-h scores), (2) the social interaction deviance composite (SIDC, sum of a–d scores minus sum of e, h, i, and j scores), (3) Pragmatic and Social Interaction Subscales (sum of e, h, i, and j scores), (4) structural language abilities (sum of a–d scores), and (5) current autistic behaviour (sum of i and j scores). Standard subscales were based around a mean of 10 and a standard deviation of 3. See Norbury et al. (2004) for details on CCC-2 validation.

### Classifying Bilinguals and Monolinguals

If caregivers indicated their child had speaking and understanding skills in English only, the child was classified as a monolingual.

Alternatively, if caregivers indicated that the child could speak and/or understand English and any other additional language(s) even with little ability, the child was classified as a bilingual. It is important to note that bilinguals in this study represent a heterogeneous sample in terms of the language combinations and respective skills in each of the languages. Furthermore, some of the bilingually classified children were in fact multilingual. Please see the “Results” section and supplementary materials for detailed description of the sample.

### Classifying High and Low Autistic Traits

**Fig. 1:**
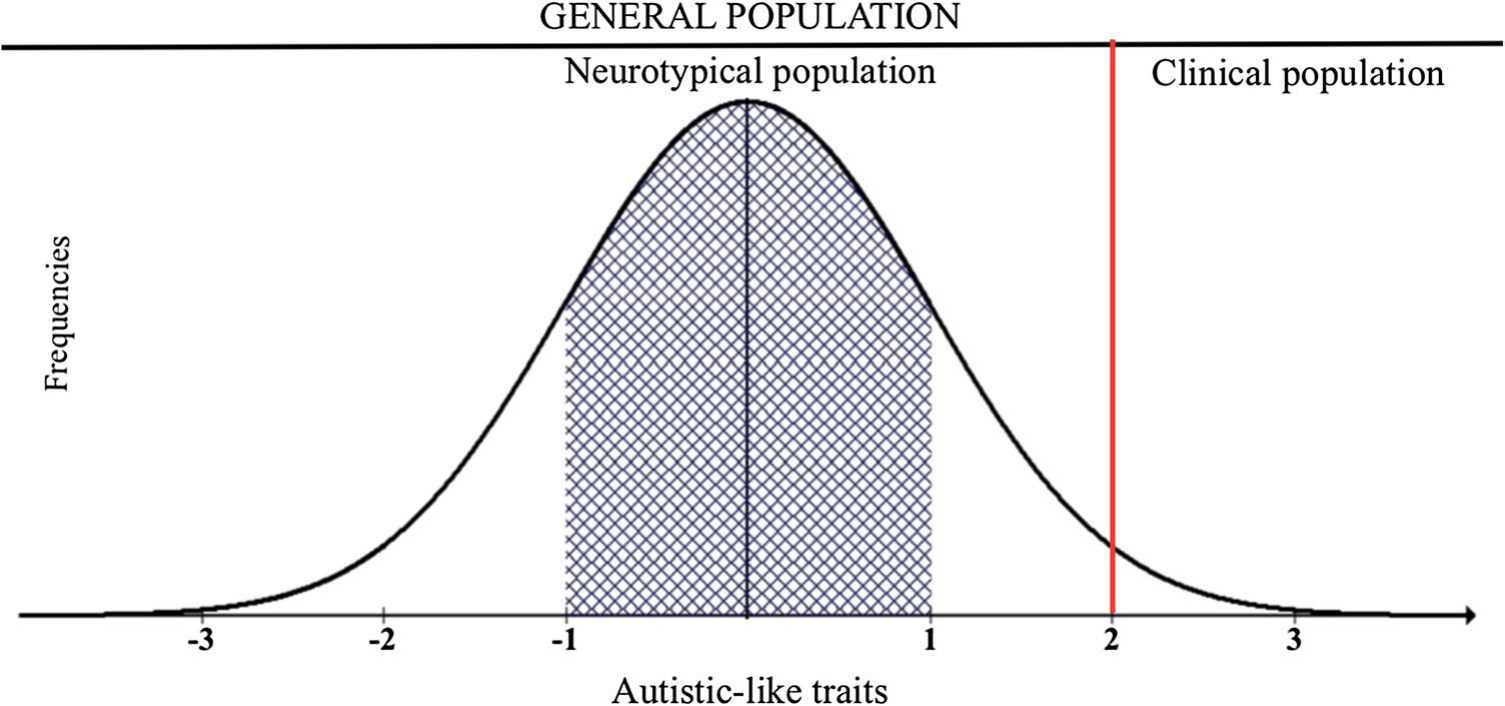
A model of the distribution of autistic-like traits in the general population in standard deviations (SD)

We used the notion of autistic-like traits (ALTs) to address our research questions. This is based on the idea that individuals can be placed on a continuum representing the extent to which they have characteristics associated with autism (Baron-Cohen et al. 2001; Burnett and Jellema 2013). Figure 1 illustrates this idea using a hypothetical probability distribution of ALT scores in a general population (GP) sample. The y axis represents the probability of obtaining a given score on a measure of ALTs (shown in standard deviations (SD) on the x axis). Most individuals in the GP score within ± 1 SD of the mean and thus could be considered ‘average scorers’, while those − 1SD from the mean are below average (‘low-scorers’), and those scoring above + 1SD from the mean are ‘high-scorers,’ who exhibit above average ALT scores. At the upper extreme of the distribution, > +2SD, this extent of ALTs could be considered similar to that expected in populations with a diagnosis of autism. In the current study, following the approach by Gresham and Elliott (2008), we use the SSIS-RS autism spectrum subscale as our measure of autistic-like traits.^2^

### Data Analysis

To answer research questions 1 and 2, we standardised SSIS-RS autism spectrum subscale scores using z-scores, then selected groups of ‘below average’ (− 1SD) and ‘above average’ (+ 1SD) children. Standard scores from the manual were not used as these are not normed using a UK population.

For research question 3, we used the SSIS-RS autism spectrum subscale scores for the whole sample as a continuous outcome variable. The third research question was addressed with the whole sample. In the first set of regression analyses, we examined the impact of some baseline factors on the distribution of autistic-like traits. Specifically, chronological age was entered in the analysis in order to account for the wide age range of our participants. Next, sex was included due to the observed bias of identifying ALTs in males. SES was also incorporated in the analysis due to recent suggestions that it might affect the distribution of ALTs (Skylark and Baron-Cohen 2017). Following this, structural language skills were added as a predictor, due to previous findings that these skills account for some variability in ALT scores distribution (Whitehouse et al. 2008). Finally, language status (bilingual vs. monolingual) was included as the last predictor to test if bilingualism accounts for any variability in the ALTs. If bilingualism improves areas of language and cognition that are often impaired in ASD, we aim to test if any variance in the ALT scores will be explained by the language status of the sample. These analyses are presented in the “Predicting Variance in ALTs”.

Apart from the binary approach regarding language status, a set of regression analyses was run separately for bilinguals so as to consider different aspects of bilingualism, such as language exposure, proficiency and literacy in both languages, and use of both languages (research question 4). These analyses are presented in the “Bilinguals and ALTs”.

## Results

### Participants and Descriptive Statistics

Four hundred and one questionnaire packs were returned. The data from 7 children were excluded for one of the following reasons: (a) incomplete SSIS-RS which included the measure of ALTs (4 children), (b) inability to deduce whether the participant is bilingual or monolingual due to missing data from the Language Use and SES Questionnaire (1 child), (c) unsigned consent form (1 child), (d) the participant was not attending the primary school and was under the age of five (1 child). This left data from 394 children for the analysis. Children who had any clinical diagnosis, intellectual, or learning difficulties were kept in the sample as our aim was to look at a general population sample of children. This approach further contributes to the ecologic validity of our findings. Concerning diagnoses or suspected diagnoses, the following were reported in the bilingual sample: global developmental delay (n = 1), suspected attention deficit/hyperactivity disorder (ADHD) and suspected to be on the spectrum (n = 1), dyslexia (n = 1), possible ADHD (n = 1), possible dyslexia (n = 1). The following diagnoses or suspected diagnoses were reported in the monolingual sample: dyslexia (n = 8), global developmental delay with autistic tendencies (n = 1), autism (n = 1), sensory processing disorder and ADHD (n = 1), developmental delay (n = 2), ADHD (n = 1), social communication disorder and autistic traits (n = 1), epilepsy (n = 1), microdeletion syndrome (n = 2), currently being assessed for autism (n = 1).

There were 230 monolinguals (121 female) with mean age of 8;4^3^ (*SD* = 1;7, age range 5;1–11;8) and 164 bilinguals (92 female) with mean age of 8;1 (*SD* = 1;7, age range 5;3–11;4). In the bilingual group, each child spoke English and (a) one other language (n = 119), (b) two other languages (n = 35), (c) three other languages (n = 8), or (d) four other languages (n = 2). Table 1 summarizes descriptive statistics of the whole sample. Note that for the bilingual group, only English and one other language (Language A) are summarised in this table. In cases of children speaking more than two languages, in Table 1 we report the data of the two most dominant ones only (one of which always happened to be English). However, when calculating the *language use with caregivers* coefficient, we compared the use of English against all other reported languages together. Please see supplementary materials for further details on the bilingual (i.e., multilingual) group.

**Table 1:** Age, language exposure, language skills, and language use of the participants

Variable	Group	n	Mean	*SD*	t-test (*p* value)
Chronological age	Bilinguals	164 (92 female)	8;1	1;7	W = 17,277 (*p* = 0.15)
	Monolinguals	230 (121 female)	8;4	1;7	
Length of residence in the UK	Bilinguals	160	6;9	2;7	W = 12,768 (*p* < 0.001)
	Monolinguals	230	8;2^b^	1;11	
Length of exposure to English	Bilinguals	164	6;10	2;6	W = 12,474 (*p* < 0.001)
	Monolinguals	230	8;4^c^	1;7	
Length of exposure to language A^a^	Bilinguals	161	7;1	2;6	
Speaking English (1–5 scale)^a^	Bilinguals	164	4.78	0.54	
Speaking language A (1–5 scale)^a^	Bilinguals	164	3.12	1.55	
Understanding English (1–5 scale)^a^	Bilinguals	164	4.86	0.38	
Understanding language A (1–5 scale)^a^	Bilinguals	164	3.54	1.51	
Writing English (1–5 scale)^a^	Bilinguals	164	4.18	0.93	
Writing language A (1–5 scale)^a^	Bilinguals	160	2.15	1.22	
Reading English (1–5 scale)^a^	Bilinguals	164	4.62	0.76	
Reading language A (1–5 scale)^a^	Bilinguals	159	2.51	1.38	
Language use with caregivers^a^	Bilinguals	160	0.68	0.23	

^a^Data available only for bilinguals

^b^For English monolinguals, it was assumed that the length of residence in the UK was from birth, unless otherwise indicated

^c^For English monolinguals, it was assumed that the exposure to English started at birth

Considering that the data for the whole sample (presented in Tables 1, 2) was not normally distributed as indicated by Shapiro–Wilk test, comparison between bilinguals and monolinguals was run by using Mann-Whitney-Wilcoxon Test. As reported in Table 1, bilinguals and monolinguals in this study did not differ in chronological age. However, monolinguals had significantly higher length of exposure to English (W = 12,474, *p* < 0.001) and higher length of residence in the UK (W = 12,768, *p* < 0.001) than bilinguals. These differences remained significant even after running a Bonferroni correction (both *ps* < 0.001). Considering language skills of bilinguals, their scores for English speaking, understanding, writing and reading skills tended to be higher than the same skills in their Language A (W = 21,676, *p* < 0.001; W = 20,427, *p* < 0.001; W = 23,164, *p* < 0.001; W = 23,026, *p* < 0.001 respectively). Furthermore, based on the *language use with the caregivers* coefficient (0.68), it seems that English was more commonly used than their other language(s). All of the differences in language skills remained significant after adding a Bonferroni correction (all *ps* < 0.001). Consequently, it can be concluded that bilinguals in this study were early English dominant bilinguals.

**Table 2:** Descriptive statistics per language group: socioeconomic status (SES), autistic-like traits (from Social Skills Improvement System-Rating Scales [SSIS-RS]), communication scores (from Children’s Communication Checklist [CCC-2])

Variable	Group	n	Mean	*SD*	t-test (*p* value)
Socioeconomic status (SES) general score (1–9 scale)^a^	Bilinguals	164	6.79	1.49	W = 18,160 (*p* = 0.56)
	Monolinguals	229	6.92	1.39	
Education for caregiver 1 (1–5 scale)^a^	Bilinguals	164	3.42	1.19	W = 24,030 (*p* < 0.001)
	Monolinguals	229	2.83	1.16	
Education for caregiver 2 (1–5 scale)^a^	Bilinguals	152	3.28	1.3	W = 18,839 (*p* = 0.01)
	Monolinguals	215	2.93	1.33	
SES composite z-score (average of the above three)^a^	Bilinguals	164	0.11	0.8	W = 22,610 (*p* < 0.001)
	Monolinguals	230	− 0.1	0.7	
Autistic-like traits raw score^b^	Bilinguals	162	8.01	5	W = 17,881 (*p* = 0.64)
	Monolinguals	227	8.47	5.7	
General communication score^c^	Bilinguals	159	81.61	20.12	W = 17,304 (*p* = 0.69)
	Monolinguals	223	82.21	19.66	
Social interaction deviance composite^c^	Bilinguals	159	− 0.33	7.95	W = 19,341 (*p* = 0.12)
	Monolinguals	223	− 1.41	8.12	
Pragmatic and social interaction^c^	Bilinguals	160	39.96	10.27	W = 18,032 (*p* = 0.91)
	Monolinguals	224	39.66	10.53	
Structural language^c^	Bilinguals	160	40.25	11.02	W = 17,169 (*p* = 0.52)
	Monolinguals	223	41.19	10.33	
Current autistic behaviour^c^	Bilinguals	160	19.69	5.46	W = 18,196 (*p* = 0.79)
	Monolinguals	224	19.45	5.65	
Speech^c^	Bilinguals	160	10.04	3.01	W = 17,854 (*p* = 0.94)
	Monolinguals	224	10.15	2.91	
Syntax^c^	Bilinguals	160	9.81	3.37	W = 17,920 (*p* = 1)
	Monolinguals	224	10.05	2.91	
Semantics^c^	Bilinguals	160	10.06	3.56	W = 16,314 *(p* = 0.15)
	Monolinguals	223	10.59	3.53	
Coherence^c^	Bilinguals	160	10.34	3.11	W = 18,186 (*p* = 0.80)
	Monolinguals	224	10.3	3.11	
Inappropriate initiation^c^	Bilinguals	160	10.46	3.19	W = 18,288 (*p* = 0.73)
	Monolinguals	224	10.37	3.18	
Stereotyped language^c^	Bilinguals	159	10.36	2.79	W = 17,578 (*p* = 0.82)
	Monolinguals	224	10.39	2.9	
Use of context^c^	Bilinguals	160	10.53	3.34	W = 18,606 (*p* = 0.52)
	Monolinguals	224	10.29	3.47	
Non-verbal communication^c^	Bilinguals	160	9.81	2.76	W = 17,532 (*p* = 0.71)
	Monolinguals	224	9.84	3.11	
Social relations^c^	Bilinguals	160	10.12	2.96	W = 20,006 (*p* = 0.04)
	Monolinguals	224	9.48	3.27	
Interests^c^	Bilinguals	160	9.57	3.35	W = 16,526 (*p* = 0.19)
	Monolinguals	224	9.97	3.07	

^a^Data obtained from the Language Use and SES Questionnaire

^b^Data obtained from SSIS-RS

^c^Data obtained from CCC-2

Table 2 shows scores on socioeconomic status (SES), ALTs (from SSIS-RS), and communication scores (from CCC-2).

As can be seen in Table 2, bilinguals’ caregivers had significantly better education levels than monolinguals’ (bilingual vs. monolingual caregiver 1, W = 24,030, *p* < 0.001; bilingual vs. monolingual caregiver 2, W = 18,839, *p* = 0.01). This difference yielded a significantly better SES composite z-score^4^ for bilinguals than monolinguals (W = 22,610, *p* < 0.001). On the CCC-2 subscale measuring social relations, bilinguals had significantly better scores than monolinguals (bilingual M = 10.12, *SD* = 2.96; monolingual M = 9.48, *SD* = 3.27; *p* = 0.04). However, after running a Bonferroni correction for multiple comparisons, only the differences in education of caregiver 1 and SES composite z-score remained significant (*p* < 0.001 and *p* = 0.01 respectively). There were no other differences between bilinguals and monolinguals.

### Bilingualism at the Extremes of Autistic-Like Traits

**Fig. 2:**
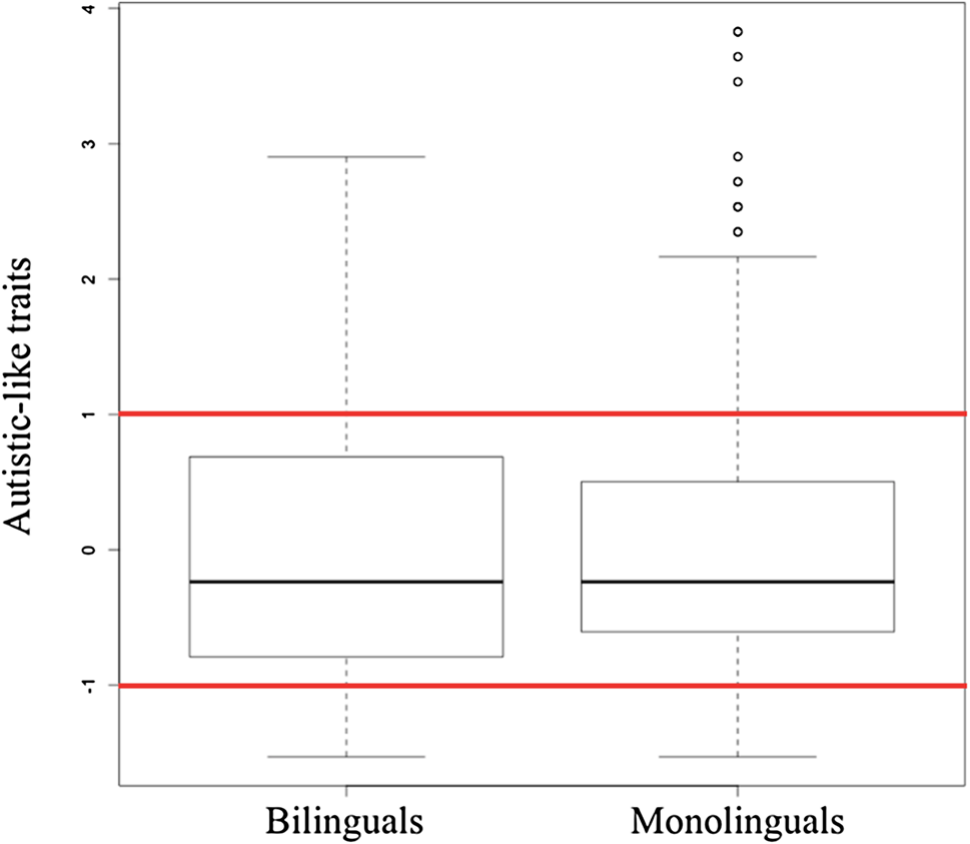
Distribution of autistic-like traits scores per language group

Our first research question asked: (1) *Is there a difference in the proportion of monolinguals and bilinguals found in groups selected from the extremes (high and low) of the autistic-like traits distribution?* Figure 2 illustrates the distribution of ALT scores in bilinguals and monolinguals with the ± 1SD cut-offs indicated with red lines. Kolmogorov–Smirnov normality test indicated that there were no differences in the distributions of autistic-like traits scores between bilinguals and monolinguals (D = 0.06, *p* = 0.87).

Among low scorers (over or equal to − 1SD), there were 22 bilinguals (41.50%) and 31 monolinguals. Among the high scorers (over or equal to + 1SD), there were 26 bilinguals (44.82%) and 32 monolinguals. There was no difference in the proportion of monolinguals and bilinguals among those exhibiting high and those exhibiting low autistic-like traits (*X*^2^ (1, *N* = 111) = 0.12, *p* = 0.72, *ns*).

The second research question, *within the high and low-scoring groups identified in (1), do bilinguals and monolinguals differ in average ALT scores?* was addressed by comparing mean ALT scores between bilinguals and monolinguals in each group.

Due to the small sample sizes in the low-scoring and high-scoring groups and the fact that the data was not normally distributed, nonparametric tests were used. A Mann-Whitney-Wilcoxon test revealed no significant differences between bilinguals (Mdn = − 1.34) and monolinguals (Mdn = − 1.16) in the low-scoring group; W = 287, *p* = 0.281, r = − 0.14. In contrast, for the high-scoring group, the ALT score was significantly lower in bilinguals (Mdn = 1.24) compared to monolinguals (Mdn = 1.61), W = 219, *p* = 0.001, r = − 0.40.

### Predicting Variance in ALTs

Our third research question asked, *what factors account for observed variance in ALT scores in a general population sample of children?*

We addressed this question using hierarchical linear regression analyses. Autistic-like traits as measured by SSIS-RS were regressed on chronological age, sex, and the SES composite at step one, structural language skills at step two, and language status (bilingual vs. monolingual) at step three (see Table 3). Finally, an additional regression analysis was run with structural language subscales (speech, syntax, semantics, coherence) instead of using the composite structural language score (see Table 4).

**Table 3:** Regression model predicting autistic-like traits for entire sample (bilinguals and monolinguals)

Variables		*β*	*∆R* ^2^	*β*	*∆R* ^2^	*β*
Step 1						
Age		− 0.11*		− 0.09*		− 0.09*
Sex		0.14**		0.06		0.06
Socioeconomic status		− 0.07		0.00		0.01
Step 2			0.32***			
Structural language skills		–		− 0.58***		− 0.58***
Step 3					0.00	
Language status (bilingual vs. monolingual)		–		–		0.07
Total *R*^2^	0.03		0.35		0.36	

*DV * Autistic-like traits

* *p* < 0.05., **p < 0.01, ***p < 0.001

**Table 4:** Regression model predicting autistic-like traits in the entire sample (bilinguals and monolinguals) with structural language subscales

Predictor variables	*β*	*p*
Age	− 0.09	0.035
Sex	0.05	0.201
Socioeconomic status	0.01	0.832
Speech	− 0.21	< 0.001
Syntax	− 0.09	0.119
Semantics	− 0.15	0.013
Coherence	− 0.24	< 0.001
Language status (bilingual vs. monolingual)	0.06	0.14

The first step significantly explained only 3% of the variance (*F*(3,374) = 4.34, *p* = 0.005). At this step, sex and age were significant predictors, such that boys had higher ALT scores than girls, and younger children had higher scores. Step two significantly improved the model (*F*(4,373) = 50.98, *p* < 0.001). At this step, sex was no longer a significant predictor, while chronological age and structural language skills explained 35% of variance in the ALT scores. Specifically, older children, and children with better structural language skills had lower ALT scores. Finally, at step three, language status was added as a predictor and this made no significant improvements to the model.

As the structural language skills measure was composed of four subscales, an additional regression analysis was run in which these subscales were entered instead of the composite score to reveal which of them contribute to the variability in ALTs. It was revealed that 36% of variance in ALT scores was significantly explained by age, speech, semantics, and coherence (*F*(8,369) = 26.34, *p* < 0.001). See Table 4.

### Bilinguals and ALTs

Our final research question asked, *considering the complexities of bilingual experiences (e.g., proficiency in both languages, use of languages), what factors account for observed variance in ALT scores in the bilingual subsample?*

In the previous model, language status was entered as a binary variable (bilingual vs. monolingual). In order to account for the diverse nature of bilingual experience (e.g., differences in proficiency, literacy, language exposure, language use; Murphy 2014), additional regression analyses were run separately for bilinguals (n = 164). Initially, Spearman correlation analyses (due to the not normally distributed data) were run between ALT scores and the following variables: chronological age, sex, SES, structural language, length of residence in the UK, length of exposure to English and Language A, language use with caregivers, proficiency^5^ in English and in Language A, literacy^6^ in English and in Language A. As can be seen in Table 5, chronological age, structural language, length of exposure to English, proficiency in English, literacy in English, and literacy in Language A significantly correlated with ALTs.

**Table 5:** Correlation analyses between bilinguals’ demographic/language data and autistic-like traits

	1	2	3	4	5	6	7	8	9	10	11	12	13
1. Age	–												
2. Sex	− 0.03	–											
3. SES	− 0.10	0.02	–										
4. Structural language	− 0.01	− 0.17*	0.12	–									
5. Length of residence	0.56***	− 0.23**	− 0.11	0.09	–								
6. Exposure to English	0.68***	− 0.04	0.02	0.15	0.70***	–							
7. Exposure to Language A	0.69***	0.07	0.01	− 0.02	0.29***	0.35***	–						
8. Use of languages with caregivers	0.08	0.06	− 0.03	0.06	0.33***	0.46***	− 0.22**	–					
9. Proficiency in English	0.22**	− 0.03	0.13	0.25**	0.37***	0.49***	0.08	0.29***	–				
10. Proficiency in Language A	0.00	− 0.07	0.13	0.10	− 0.32***	− 0.34***	0.32***	− 0.80***	− 0.15	–			
11. Literacy in English	0.62***	− 0.12	− 0.02	0.24**	0.53***	0.52***	0.33***	0.16*	0.45***	− 0.07	–		
12. Literacy in Language A	0.22**	− 0.07	0.17*	0.22**	− 0.08	− 0.02	0.30***	− 0.43***	0.01	0.66***	0.21**	–	
13. Autistic-like traits	− 0.16*	0.03	− 0.01	− 0.67***	− 0.13	− 0.20*	− 0.07	− 0.07	− 0.28***	− 0.07	− 0.28***	− 0.16*	–

*p < 0.05, **p < 0.01, ***p < 0.001

Consequently, these variables were entered as predictors in the regression model with ALT scores as the dependent variable at step one. As in the previous analyses, chronological age and structural language skills were the only significant predictors. They explained 52% of the variance in ALT scores (*F*(6,147) = 26.64, *p* < 0.001), such that older children and children with better structural language had lower ALT scores (see Table 6).

**Table 6:** Regression model predicting autistic-like traits in bilinguals

Predictor variables	*β*	*p*
Age	− 0.24	0.016
Structural language skills	− 0.69	< 0.001
Length of exposure to English	0.11	0.229
Proficiency in English	− 0.08	0.348
Literacy in English	− 0.02	0.847
Literacy in language A	0.03	0.631

In a separate regression analysis, the structural language measure was substituted by its subscales. It was found that chronological age, and the subscales of *speech, semantics*, and *coherence* explain 52% of variance in ALT scores in bilinguals (*F*(9,144) = 17.68, *p* < 0.001), (see Table 7).

**Table 7:** Regression model predicting autistic-like traits in bilinguals with structural language subscales

Predictor variables	*β*	*p*
Age	− 0.24	0.017
Speech	− 0.23	0.005
Syntax	− 0.12	0.167
Semantics	− 0.23	0.012
Coherence	− 0.24	0.008
Length of exposure to English	0.11	0.237
Proficiency in English	− 0.09	0.272
Literacy in English	− 0.01	0.954
Literacy in language A	0.04	0.598

While certain subscales of structural language skills significantly predict a part of ALTs variance in both bilinguals and monolinguals (as well as chronological age in bilinguals and sex in monolinguals), the explained portion of this variance seems to be higher in the bilingual sample (52%) than in monolinguals (29%).^7^

## Discussion

The aim of the current study was to explore the potential interplay between bilingualism and autistic-like traits in a general population sample of primary school children. Since bilingualism has been reported to have a positive impact on certain areas of communication and cognition, and since these aspects tend to be impaired in ASD, it was hypothesised that there might be differences in the extent of ALT scores between monolingual and bilingual children. Broadly speaking, our findings do not seem to support this claim. Specifically, in the ‘high-scoring’ and ‘low-scoring’ groups, based on ALTs, we found no differences in frequencies of monolinguals and bilinguals. If bilingualism had influenced the manifestation of ALTs, we would expect proportionally fewer bilinguals than monolinguals in the high traits group and proportionally more bilinguals in the low traits group. This was not the case.

We further explored this hypothesis by comparing the ALT scores of bilinguals and monolinguals in the high and low groups, as well as in the entire sample. In the low traits group, there were no significant differences in ALT scores between bilinguals and monolinguals. However, there is some tentative evidence for an effect of bilingualism on ALT scores, because bilinguals in the high scores category had significantly lower ALT scores compared to monolinguals. To explore this relation further, and to see whether it held within the whole sample, we ran a series of regression analyses in order to check if language status (bilingual vs. monolingual) explains any of the variance in the ALT scores in the whole sample of children in our study (i.e., not just the children scoring high or low in ALTs). We found that language status had no effect. However, factors that seems to explain a large portion of variance in the ALT scores in addition to chronological age were structural language skills, in particular, speech, semantic knowledge and coherence.

Due to the heterogeneity of bilingual experiences (see Murphy 2014), follow-up analyses took a more detailed approach instead of treating language status as a binary variable. Therefore, separate regressions were run for bilinguals to determine a potential role of language proficiency, literacy, exposure, and language use. These analyses showed that no aspect of bilingualism significantly explains variance in autistic-like traits in this sample. There are several potential explanations for this pattern of results.

First, it is possible that there is no meaningful relation between bilingualism and ALTs at all. The significant difference in ALT scores of bilinguals and monolinguals in the high scoring group might potentially be due to some other factor, e.g. the relatively small number of participants in the high scoring group (monolinguals n = 32, bilinguals n = 26) compared to the whole sample (n = 394). This is the most conservative interpretation of our findings and it is the one we tend to adopt until and unless further research reveals novel evidence.

That being said, it is interesting at this early stage of research in this important area to consider alternative reasons why we did not find convincing evidence to reject the null hypotheses concerning the relation between bilingualism and ALTs. One possibility is that this is because bilinguals in this study were English-dominant. It could be the case that the relation between bilingualism and ALTs would show more clearly in bilingual communities where both languages are used in a more balanced way (this is likely to be the case in settings where two or more languages have a similar status, e.g., when they are officially recognized by the state and when the community has positive attitudes towards the languages in question). It is plausible that this balance would lead to more opportunities for using the inhibitory control and ‘mind-reading’ skills needed to switch languages frequently and appropriately (Bak 2016).

Additionally, a link between bilingualism and ALTs might be more evident with more bilingual experience. As Luk et al. (2011) suggest, apart from the early age of becoming bilingual, prolonged bilingual experience contributes to the cognitive advantages of bilinguals. If these advantages in cognitive skills, which are often impaired in ASD, are more evident with prolonged bilingual exposure, investigating the relationship between bilingualism and ALTs in adults and the elderly requires attention. Studies indicating the attenuating effect of bilingualism on cognitive decline in the elderly support this suggestion (see Bialystok et al. 2004). The current study explored the relation between bilingualism and ALTs in children who have had a relatively limited exposure to two languages. However, adults with a longer or life-long exposure to two languages might show potential links between bilingualism and ALTs.

Another tentative explanation for our findings is that the potential link between bilingualism and ALTs might be evident only in extreme cases, that is, in groups with very high ALT scores and in clinical groups with ASD. In particular, positive effects of bilingualism (if existent) might be weak, and only evident in those situations when social skills and other relevant behaviours are particularly depressed due to the high presence of autistic symptomatology. Since there was no clinical group in our study, and the questionnaires used in this study were not diagnostic instruments, the above claim could not be verified in our dataset. However, the finding that in the high scoring group there were lower ALT scores for the bilinguals compared to the monolinguals is some indicative evidence for this hypothesis. Additional evidence to this effect is reported by Gonzalez-Barrero and Nadig (2017), who found an advantage for bilinguals with ASD in executive functions.

Turning to the role of structural language in predicting variance in ALT scores, the findings of this study support the outcome in Whitehouse et al. (2008) and Eigisti et al. (2011), who found a link between structural language skills and ASD traits in clinical samples. We extend these findings to a general population sample, as well as to the bilingual population, and find that lower levels of structural language skills seem to be linked to higher ALT scores. We also observe that these skills play a different role in bilinguals and monolinguals. Specifically, while sex and certain structural language skills (speech and coherence) explained 29% of variance in ALTs among monolinguals, chronological age and structural language (speech, semantics, and coherence) explained 52% of variance in ALTs among bilinguals. Note that bilinguals and monolinguals did not differ on the baseline structural language skills measures (see “Participants and Descriptive Statistics”, Table 2). We also note that our study identifies no differences between bilinguals and monolinguals on any of the communication skills measures. This finding corroborates Reetzke et al.’s (2015) study with a clinical sample, which identified no differences between bilingually and monolingually exposed children in communication skills as measured by CCC-2.

Our findings on structural language skills have implications for speech and language therapists, as well as for theories of language development in ASD. Specifically, the findings suggest that the importance of structural language in relation to ALTs is higher in bilinguals even when they do not lag behind monolinguals’ language development milestones. Research among clinical groups has already identified distinct language phenotypes in ASD and suggested demarcating individuals with and without language impairment among the autistic population (Kjelgaard and Tager-Flusberg 2001; Norbury 2014a, b).^8^ Future studies should therefore explore this distinction in both bilinguals and monolinguals.

Although our analysis identifies the link between structural language skills and ALTs, conclusions about causality cannot be made based on our data. Future work needs to determine the nature of this link. A first step would be to use more detailed instruments for assessing autistic-like traits and to take up a more qualitative approach in determining the areas of impairment/strengths rather than relying on a composite ALT score as we did in our study. This could be done by making clear distinctions between impairments/strengths in social communication, social interaction, restricted interests, and repetitive behaviour for each participant.

Second, there is a limitation in our study regarding the use of CCC-2 for measuring communicative skills. Specifically, in the case of bilinguals, there is a possibility that caregivers rated their children’s communicative abilities considering both languages together rather than English only. However, as the caregivers were not sent additional instructions for filling in the questionnaire apart from the ones contained in the standard CCC-2, we can assume that they rated their children’s communication skills in English only rather than in both languages. This is due to the fact that CCC-2 illustrates the phenomena it measures only with examples in English (e.g., ‘Leaves off beginnings or ends of words, e.g. says “roe” instead of “road” or “nana” instead of “banana”’).

Additionally, direct measures of structural language skills and language proficiency in general could offer more accuracy. Specifically, in our study, caregivers’ reports were distributed only in English. As some bilingual parents do not speak English as their first language, there is a chance that their English proficiency could have affected their responses. For this reason, all caregivers were provided with contact details of the authors in case they needed clarifications regarding the questionnaires. Furthermore, a body of literature has identified parental reports of language skills to be a reliable measure of linguistic ability in both monolingual and bilingual contexts, as well as in neurotypical and non-neurotypical children (Dale 1991; Thal et al. 1999; Paradis et al. 2010; Miller et al. 2017). However, more accuracy regarding language skills and areas of difficulty can be obtained by using a combination of a parental report and direct measures of children’s structural language skills (Dale 1991; Boerma and Blom 2017). Additionally, Lee et al. (2009) suggest that teacher reports further contribute to obtaining a more accurate picture of children’s language abilities. This is of particular relevance for bilingual children who are often exposed to one language at home and to another one at school. Therefore, future studies should triangulate data from different sources (e.g. a parent report, direct assessment, a teacher report) in order to gain a more comprehensive view of language abilities across contexts and languages and determine the role that structural language plays in the variability of ALTs.

Finally, as Norbury (2016) suggests, longitudinal data is required in order to establish the ways in which language learning/development and certain impairments in ASD are linked. Specifically, tracking bilinguals’ and monolinguals’ language development as well as measuring their ALTs longitudinally can help answering the question of how structural language skills and ASD symptomatology interact. This, however, sets additional challenges for researchers aiming to collect large data samples considering financial and time burdens of testing and tracking participants individually over a longer period.

Overall, the current study provides the first explorations of the interplay between bilingualism and autistic-like traits in a general sample of primary school children in the UK. While this study provides preliminary evidence of the interaction between structural language skills and ALTs in both bilinguals and monolinguals, further work is required. We hope that follow-up studies will extend this investigation to clinical samples and other bilingual contexts including adult populations as well as children. Furthermore, more sensitive instruments measuring ALTs as well as direct measures of language skills and teacher reports (rather than only caregivers’ reports) could offer more reliable evidence of potential links between bilingualism and ASD symptomatology.

## Electronic supplementary material

Below is the link to the electronic supplementary material.

Supplementary material (PDF 272 KB)

## Footnotes

1 In this paper, we use the distinction between gender and sex as outlined by the World Health Organization (WHO): “Gender refers to the socially constructed characteristics of women and men—such as the norms, roles and relationships that exist between them. […] While most people are born either male or female (biological sex), they are taught appropriate behaviours for males and females (gender norms) […].” (World Health Organization 2015).

2 Note, however, that the questionnaire used in this study to quantify autistic-like traits is not a diagnostic tool. It simply measures autistic-like traits in a general population. High scores on this scale would require attention and further assessment by qualified clinicians, but they would by no means imply diagnosis of ASD.

3 8 years and 4 months.

4 Calculated by averaging SES general score, education of caregiver 1, and education of caregiver 2 z scores.

5 A sum score of speaking and understanding skills in English and Language A respectively.

6 A sum score of writing and reading skills in English and Language A respectively.

7 A separate regression model was run for monolinguals with chronological age, sex, SES, and the structural language (subscales) as predictors, and showed that sex (β = 0.12, *p* = .04), *speech* (β = − 0.18, *p* = .02), and *coherence* (β = − 0.26, *p* = .001) account for 29% of variance in ALTs (*F*(7,212) = 12.51, *p* < .001).

8 However, see Whitehouse et al. (2008) who warn against equalising language profiles of autistic individuals with language impairments to those of individuals with specific language impairments.
