# Supplementary material for: Relations Between Bilingualism and Autistic-Like Traits in a General Population Sample of Primary School Children
Source: J Autism Dev Disord. 2019 Apr 11;49(6):2509–23. doi: 10.1007/s10803-019-03994-2 (PMC6546658; doi:10.1007/s10803-019-03994-2)
Supplement: Supplementary file 1 — Supplementary material (PDF 272 KB) [file 10803_2019_3994_MOESM1_ESM.pdf]

Supplementary Materials

Article title: Relations between bilingualism and autistic-like traits in a general population sample of primary school children

Journal name: Journal of Autism and Developmental Disorders

Author names: 1. Draško Kašćelan[1]; 2. Napoleon Katsos[1]; 3. Jenny L. Gibson[2]

Affiliations:

[1]Faculty of Modern and Medieval Languages, University of Cambridge (Raised Faculty Building, Sidgwick Avenue, Cambridge, CB3 9DA)

[2]Faculty of Education, University of Cambridge (184 Hills Road, Cambridge, CB2 8PQ)

The email address of the corresponding author: kascelandrasko@gmail.com; dk497@cam.ac.uk

Information about proficiency, exposure, and frequency to Language B, Language C, and Language D of multilinguals in the study

Table 1 *Number of languages reported in the multilingual group*

Number of languages reported	n
2 languages reported (Eng + Lang A)	119
3 languages reported (Eng + Lang A + Lang B)	35
4 languages reported (Eng + Lang A + Lang B + Lang C)	8
5 languages reported (Eng + Lang A + Lang B + Lang C + Lang D)	2
**Total participants**	**164**

Table 2 *Proficiency ratings for Language B*

Ratings for Language B			
**Variable**	**n**	**Mean**	***SD***
Speaking Language B (1-5 scale)	45	2.31	1.31
Understanding Language B (1-5 scale)	45	2.73	1.44
Writing Language B (1-5 scale)	44	1.8	0.98
Reading Language B (1-5 scale)	44	2.23	1.2
Length of exposure to language B (years;months)	44	5;11	2;10

Table 3 *Proficiency ratings for Language C*

Ratings for Language C			
**Variable**	**n**	**Mean**	***SD***
Speaking Language C (1-5 scale)	9	1.56	0.73
Understanding Language C (1-5 scale)	9	2.33	1.41
Writing Language C (1-5 scale)	9	1.44	0.73
Reading Language C (1-5 scale)	9	1.78	0.83
Length of exposure to language c (years;months)	9	5;9	2;9

Table 4 *Proficiency ratings for Language D*

Ratings for Language D
Data not collected

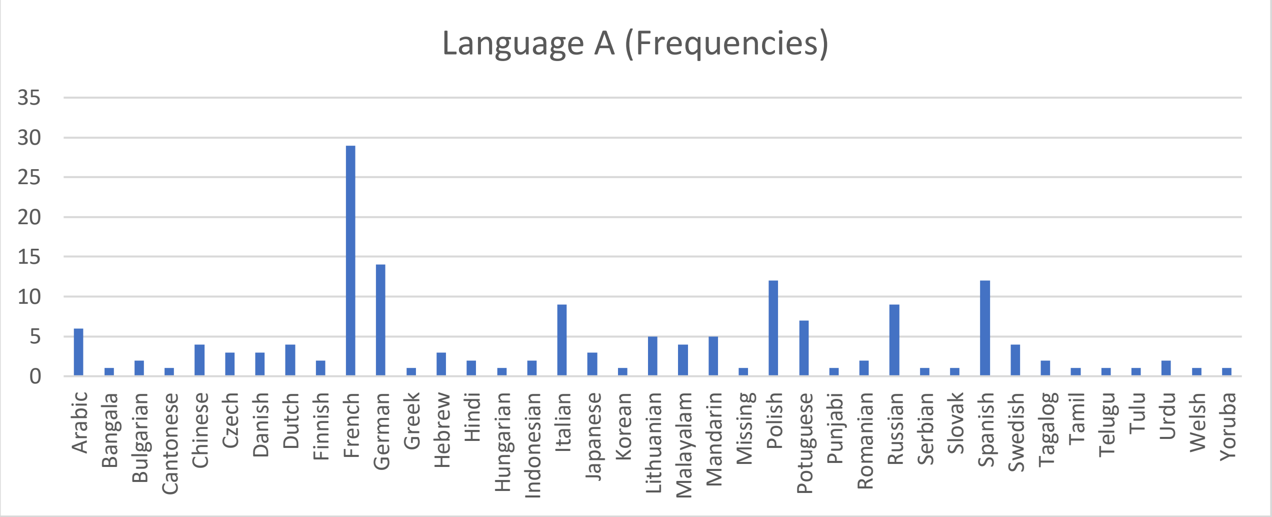

Fig. 1 Frequencies of Language A in the multilingual group

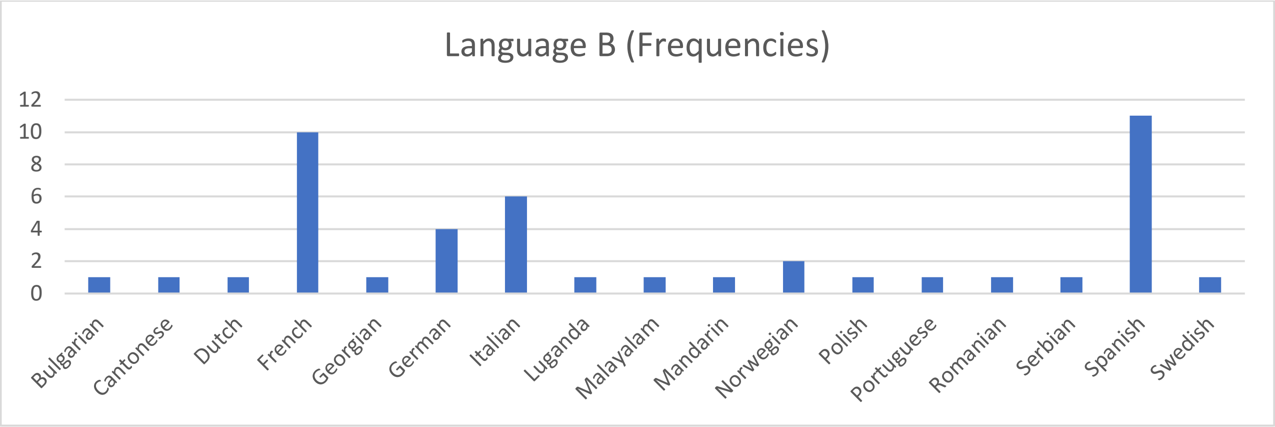

Fig. 2 Frequencies of Language B in the multilingual group

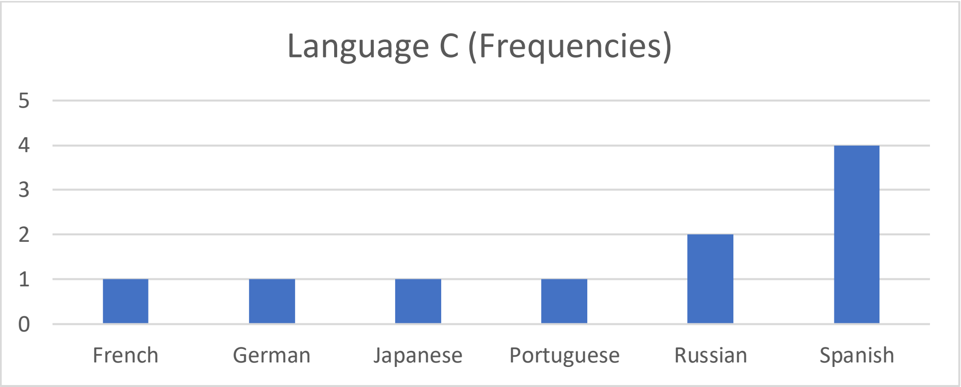

Fig. 3 Frequencies of Language C in the multilingual group

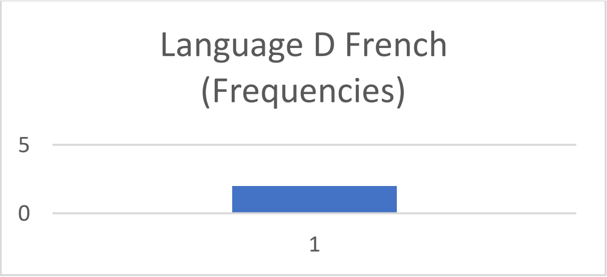

Fig. 4 Frequencies of Language D in the multilingual group
